# Safety of Digestive Endoscopy following Acute Coronary Syndrome: A Systematic Review

**DOI:** 10.1155/2016/9564529

**Published:** 2016-03-10

**Authors:** Alastair Dorreen, Sarvee Moosavi, Myriam Martel, Alan N. Barkun

**Affiliations:** ^1^Department of Internal Medicine, McGill University, Jewish General Hospital, Montreal, QC, Canada; ^2^Division of Gastroenterology, The McGill University Health Center, Montreal General Hospital Site, 1650 Cedar Avenue, Room D7-185, Montreal, QC, Canada H3G 1A4; ^3^Epidemiology Biostatistics and Occupational Health, McGill University Health Centre, McGill University, Montreal, QC, Canada

## Abstract

*Background.* The safety of endoscopy after an acute coronary syndrome (ACS) is poorly characterized. We thus performed a systematic review assessing the safety of endoscopy following ACS.* Methods.* Searches in EMBASE, Medline, and Web of Science identified articles for inclusion. Data abstraction was completed by two independent reviewers.* Results.* Fourteen retrospective studies yielded 1178 patients (mean 71.3 years, 59.0% male) having suffered an ACS before endoscopy. Patients underwent 1188 endoscopies primarily to investigate suspected gastrointestinal bleeding (81.2%). Overall, 810 EGDs (68.2%), 191 colonoscopies (16.1%), 100 sigmoidoscopies (8.4%), 64 PEGs (5.4%), and 22 ERCPs (1.9%) were performed 9.0 ± 5.2 days after ACS, showing principally ulcer disease (25.1%; 95% CI 22.2–28.3%) and normal findings (22.9%; 95% CI 20.1–26.0%). Overall, 108 peri- and postprocedural complications occurred (9.1%; 95% CI 7.6–10.9%), with hypotension (24.1%; 95% CI 17.0–32.9%), arrhythmias (8.1%; 95% CI 4.5–18.1%), and repeat ACS (6.5%; 95% CI 3.1–12.8%) as the most frequent. All-cause mortality was 8.1% (95% CI 6.3–10.4%), with 4 deaths attributed to endoscopy (<24 hours after ACS, 3.7% of all complications; 95% CI 1.5–9.1%).* Conclusion.* A significant proportion of possibly endoscopy-related negative outcomes occur following ACS. Further studies are required to better characterize indications, patient selection, and appropriate timing of endoscopy in this cohort.

## 1. Introduction

Performing endoscopic procedures in the setting of an acute coronary syndrome (ACS) can prove challenging. These patients are at increased risk of arrhythmias, heart failure, further ischemic events, and death [1, 2]. The stress of undergoing endoscopic procedures with the utilization of procedural sedation can theoretically precipitate cardiac complications and increase procedural risk.

As a result of these concerns, physicians may be hesitant to perform endoscopy following an ACS. Currently, there exists no consensus regarding the optimal timing of an urgent endoscopy following an ACS. We present a systematic review of the literature on the safety, efficacy, and complications of luminal endoscopy in this setting.

## 2. Methods

### 2.1. Review of the Literature

A comprehensive computerized medical literature search was performed using MEDLINE, EMBASE, Cochrane library, and the ISI Web of Knowledge from 1990 to April 2014. A highly sensitive search strategy was used to identify all observational studies (case-control, cohort, or case series) with a combination of controlled vocabulary (MeSH) and text words related to (1) upper or lower gastrointestinal endoscopy or ERCP and (2) myocardial infarction or acute coronary syndrome (in the appendix).

All adult human studies in English were included as well as published abstracts from scientific meetings only if the data were not duplicated in subsequent publications. Recursive searches and cross-referencing were also carried out using a “similar articles” function; hand searches of articles were identified after an initial search.

### 2.2. Study Inclusion and Patient Population

Two authors independently reviewed all abstracts for potential inclusion. Relevant abstracts were then further reviewed based on manuscript content, with a third independent reviewer resolving any disagreements.

Case reports were excluded, and case series with a sample size of <5 were also excluded. Papers published by the same author(s) were screened for duplication of results. In order to be included, publications had to have assessed patients undergoing endoscopy within 60 days of suffering an ACS. ACS was defined as unstable angina, non-ST elevation myocardial infarction (NSTEMI), ST elevation myocardial infarction (STEMI), or ACS not specified. All forms of endoscopic diagnostic or therapeutic procedures involving the gastrointestinal tract were considered for inclusion into the study.

### 2.3. Data Collection and Statistical Analysis

Information from all relevant papers, including demographic information, type of endoscopy, indications, complication rates, and ACS subtypes, was compiled. Complications were defined based on initial descriptions provided in the papers being reviewed. Complications were subsequently classified according to general categories. Two authors independently classified complication events as major or minor. For every study, we determined weighted data for timing of endoscopy and rates of endoscopic complications and all-cause mortality. Descriptive statistics included categorical variables expressed as proportions and 95% confidence intervals and continuous variables as means ± standard deviation or medians and ranges. All statistical analyses were performed using SAS 9.2, SAS Institute Inc., Cary, NC, USA.

## 3. Results

### 3.1. Identified Citations

**Figure 1:**
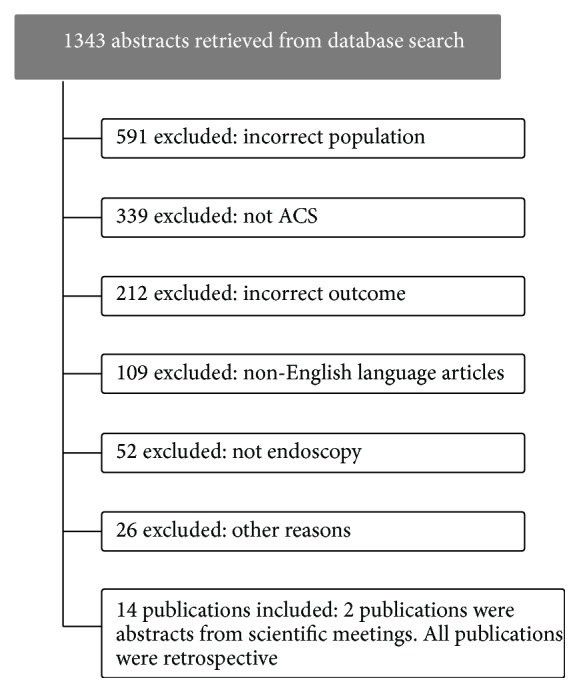
STROBE diagram.

Initial search of the databases yielded 1343 citations. After review, 1329 were excluded for the following reasons: incorrect outcome, non-English language article, and incorrect population (STROBE diagram, Figure 1). Fourteen publications [3–16] were included in the analysis, 2 of which were abstracts from scientific meetings; all publications were retrospective cohorts. The publication dates ranged within 1993–2014.

### 3.2. Patient Population

Overall, 1178 patients suffering from a recent ACS underwent 1188 endoscopies. The mean age was 71.3 ± 3.8 years, and 59.0% were male. The incidence of endoscopy following an ACS was 0.48% (data on 274/56,674, Table 1). All patients had suffered an ACS (59.8% NSTEMI, 20.2% STEMI and nonspecified in 19.9%). A third of patients developed congestive heart failure (32.9%) and 19.4% developed arrhythmias secondary to ACS before endoscopy; 18.2% were ventilated at the time of endoscopy (Table 1).

### 3.3. Endoscopic Procedures

Procedures included 810 EGDs, (68.2%), 191 colonoscopies (16.1%), 100 sigmoidoscopies (8.4%), 64 PEGs (5.4%), and 22 ERCPs (1.9%). The primary indications for endoscopy were unspecified symptoms of gastrointestinal (GI) bleeding (28.8%), occult blood loss/anemia (20.3%), hematemesis (14.2%), melena (12.7%), hematochezia (5.3%), and other indications (18.8%) (Table 2). The average timing to endoscopy was 9.0 ± 5.2 days after ACS; sedation was used in 87.0% (95% CI 84.2–89.3%) of endoscopies (Table 2). The most common endoscopic findings were peptic ulcer disease (25.1%; 95% CI 22.2–28.3%), followed by normal endoscopic findings (22.9%; 95% CI 20.1–26.0%). Gastritis and esophagitis accounted for 20.5% (95% CI 18.8–23.4%) of findings (Table 3). Of all endoscopies performed, 20.2% (95% CI 17.5–23.2%) were therapeutic.

### 3.4. Outcomes

Outcomes following initial endoscopy included 10 patients requiring repeat endoscopy (2.2%; 95% CI 1.2–4.0%). Following endoscopy, 3.6% of patients (95% CI 2.2–4.8%) required gastrointestinal surgery or angiography for ongoing management. Including deaths attributable to endoscopy, 108 complications occurred across all endoscopic modalities (9.1%; 95% CI 7.6–10.9%). Of all complications, 72.4% were classified as major (95% CI 63.2–80.0%, data on 105), accounting for a major complication rate of 6.4% (95% CI 5.2–8.0%). The complication rate for the different endoscopic modalities was 11.5% (95% CI 9.2–14.4%) for EGD, 9.0% (95% CI 4.8–16.2%) for colonoscopy, 2.5% (95% CI 0.7–8.7%) for flexible sigmoidoscopy, 10.3% (95% CI 3.6–26.4%) for PEG, and 14.3% (95% CI 5.0–34.6%) for ERCP (Table 4). When the overall complication rate was broken down by type of complication, hypotension (24.1%; 95% CI 17.0–32.9%), arrhythmias (8.3%; 95% CI 4.5–15.1%), and repeat ACS (6.5%; 95% CI 3.2–12.8%) accounted for the majority of complications encountered across all endoscopic modalities (Table 5). Four deaths were attributed to endoscopy (<24 hours after ACS, 3.7% of all complications; 95% CI 1.5–9.1%). Of these 4 deaths, 1 was temporally related, occurring 14 hours after procedure [10], with the cause of death remaining unknown. The remaining 3 deaths were from fatal arrhythmias, reported as occurring intraprocedurally. All-cause mortality was 8.1% (95% CI 6.2–10.1%).

## 4. Discussion

There is a paucity of data surrounding the safety of endoscopy following ACS. This stems from the unpredictable nature, varying indication, and low incidence of endoscopy after ACS. This systematic review was performed to summarize existing information to guide clinicians in future management of their patients. The primary outcome of this study revealed an overall complication rate for all endoscopic procedures to be 9.1% (95% CI 7.6–10.9%), suggesting that this group of patients is prone to adverse events related to endoscopy. Even in the absence of recent ACS, endoscopy has been documented to provoke silent ischemic changes in patients with underlying coronary artery disease [17–19]. When the indication for endoscopy is acute GI bleeding, those patients with coronary artery disease (CAD) are at increased risk for both silent ischemia and arrhythmias [20]. The adrenergic response to endoscopy is felt to act as a “stress test,” provoking ischemia in a supply-demand fashion.

The close proximity of endoscopy to initial ACS (mean 9.0 ± 5.2 days) raises concerns about increased cardiopulmonary complications. The important issue of elapsed time between the ACS and endoscopy with regard to incidence of complications was only assessed in one study. Indeed, Spier et al. noted an inverse relationship between endoscopic complications from timing of ACS; in 135 patients, the only 2 complications occurred with endoscopy performed the same day as ACS, in contrast to no complications in procedures performed 24 hours or more after an ACS [8].

Sharma et al. recently looked at all GI endoscopies entered into the Clinical Outcomes Research Initiative database over a 5-year period to identify factors predictive of endoscopic complications [21]. Conscious sedation, the age of the patient, a higher American Society of Anaesthesiology (ASA) grade, inpatient status, trainee participation, and routine use of oxygen were all associated with unplanned cardiopulmonary adverse events during GI endoscopy [21]. Many of the patients with recent ACS share these characteristics, suggesting that the additive role of recent injury must infer higher risk of cardiopulmonary complications.

The results presented here are in contrast to previous reports of complications encountered during elective endoscopy. Existing data have shown complications of upper GI endoscopy ranging from 1 in 200 to 1 in 10,000 [22–25], with recent data on mortality showing a rate of 1 in 10,000 [21]. In a large, systematic review including 57,742 patients across 17 prospective studies, serious adverse advents during screening colonoscopy were reported to occur at a rate of 2.8 complications per 1000 procedures [26]. A Canadian study looked at complications rates of 97,091 patients undergoing outpatient colonoscopy, finding 1.64 per 1000 and 0.85 per 1000 events for perforation and bleeding, respectively [27].

In contrast, in the current ACS setting, the calculated complication rates were 11.5% (95% CI 9.2–14.4%) and 9.0% (95% CI 4.8–16.2%), for EGD and colonoscopy, respectively; because of the size of the sampling, these values were felt to be more generalizable than the observed complication rates for PEG (10.3%; 95% CI 3.6–26.4%) and ERCP (14.3%; 95% CI 5.0–34.6%) that were based on smaller number of procedures (PEG *n* = 64, ERCP *n* = 22). Despite this, the high rate of complications seen with ERCP can be explained by its technical difficulty and the concomitant disease processes, largely cholangitis or choledocholithiasis (Table 2, appendix). Moreover, the increase in complications across all endoscopic procedures is attributable to the comorbid state of these patients. A third suffered from congestive heart failure and a fifth from arrhythmias in the immediate period after ACS (data on 310), and at the time of endoscopy 18.2% (95% CI 15.9–20.7%) were mechanically ventilated.

The temporal relationship of complications to endoscopy also suggests that, as part of the endoscopy itself, procedural sedation is likely a contributing factor in the majority of complications. In this review, 87.0% (95% CI 84.2–89.3%) of patients received some form of sedation for the procedure. In a prospective study including 17,999 endoscopic procedures, 96% of patients received propofol as the primary sedative with an observed sedation-related complication rate of 4.51% [28]. This is contrasted to older data describing the use benzodiazepines in 93% of 21,011 endoscopic procedures, with a sedation-related complication rate of 1.35% [29]. A similar study exhibited an even lower sedation-related complication rate of 0.10% across 115,200 procedures [22] in which benzodiazepines were the primary sedative.

In this review, the majority of patients underwent endoscopy to evaluate clinical or biochemical signs of GI bleeding (81.2%). Patients receiving standard medical therapy for ACS, namely, dual antiplatelet therapy and therapeutic low molecular weight heparin, are at risk of clinically significant GI bleeding, with a previously reported incidence of 2.7% [30]. Among all patients with ACS, the rate of overt GI bleeding is 0.7–1.3% [31, 32]. As more ACS patients are treated with primary percutaneous intervention (PCI), more are likely to suffer acute GI bleeding in the post-ACS period due to the use of high-dose intraprocedural anticoagulation and the resulting necessity for long-term antiplatelet agents. Presently, the incidence of overt GI bleeding following PCI is reported to range within 1.2–2.3% [33, 34]. Overall, patients who have GI bleeding following an ACS have a higher all-cause mortality compared to their nonbleeding counterparts [32–34] in addition to higher rates of cardiac mortality [32].

Preventative measures should be employed to decrease the need for endoscopy, including the appropriate use of gastroprotective agents for those on antiplatelet agents. The routine use proton pump inhibitors (PPI) are preferred over histamine-2 receptor antagonists (H2RA) [35]. Furthermore, concerns regarding interactions between PPI and antiplatelet agents have been deemed to be clinically invalid [36, 37]. If preventative measures fail, endoscopists should look towards improving endoscopic outcomes. Clinicians may find the use of hemostatic powders, such as TC-325, beneficial to lessen the intraprocedural time [38]. Other possibilities for improving success include deferring diagnostic and nonurgent procedural endoscopy. Based on these data, we suggest that patients having suffered a recent ACS should have procedural electrocardiographic monitoring in addition to standard monitoring. The ASA currently recommends continuous electrocardiographic monitoring for patients with history of cardiovascular disease or arrhythmias [39]. Determining the need for more intensive monitoring should be done at the discretion of the digestive endoscopist in consultation with the cardiology team.

Methodological limitations include the poor quality of the data, precluding the ability to perform a formal meta-analysis. In addition, adjudication of outcomes is not feasible given the nature of the data, limiting attribution of specific complications directly to endoscopy. The data collected are heterogeneous; the changing definition of ACS over time and the variation in the definition of complication across the articles force one to interpret the data cautiously. This highlights, however, the need for prospective data on the safety of endoscopy after ACS. Currently, these data are the best that exist regarding this topic. Finally, one author was responsible for 7 of the 19 publications included in our review, raising the possibility of selection bias. As they were felt to be of higher quality, we kept these studies, but steps were taken to ensure that we excluded duplicate data among studies.

This systematic review nonetheless clearly quantifies an increased rate of negative outcomes in ACS patients undergoing digestive endoscopy. The clinical management of ACS patients must therefore include minimizing risks such as GI bleeding. While not validated in this systematic review, the temporal relationship between the ACS event and the procedure may contribute to exacerbating cardiopulmonary complications. The risks and benefits must therefore be carefully considered on a case-by-case basis, limiting urgent endoscopy to clinical scenarios where a therapeutic benefit is likely to alter the patient course.

## Figures and Tables

**Table 1:** Patient demographics & ACS characteristics.

Article		Population					ACS			ACS complications		
Author	Year	Incidence^+^	Range	Age	Male	*N*	AMI	NSTEMI	STEMI	CHF	Arrhythmia	Ventilated
Cappell and Iacovone Jr. [3]	1996	0.241%	30	72.5	64% (18)	28	100% (28)	0	0	0	0	61% (17)
Montalvo and Lee [4]	1996	x	21	66.0	88% (7)	8	0	100% (8)	0	x	x	x
Al-Ebrahim et al. [5]	2012	x	31	73.5	45% (20)	44	100% (44)^1^	0	0	x	x	x
Nojkov and Cappell [6]^*∗*^	2010	0.250%	26	77.9	38% (5)	13	0	85% (11)	15% (2)	23% (3)	38% (5)	31% (4)
Nojkov and Cappell [6]^*∗*^	2010	x	30	73.1	67% (4)	6	100% (6)^2^	0	0	0	0	17% (1)
Mumtaz et al. [7]	2008	x	30	66.0	78% (66)	85	0	82% (70)	18% (15)	51% (43)	36% ^*∗*^(31)	16% (14)
Spier et al. [8]	2007	x	30	68.0	63% (85)	135	0	83% (112)	17% (23)	x	x	x
Lin et al. [9]	2006	0.825%	7	69.1	67% (70)	105	100% (105)	x	x	x	x	23% (24)
Cappell [10, 11]	2004	0.680%	30	73.3	62% (62)	100	2% (2)	70% (70)	28% (28)	30% (30)	10% (10)	18% (18)
Cappell [10, 11]	2004	x	30	74.0	47% (37)	78	0	64% (50)	36% (28)	33% (26)	18% (14)	33% (26)
Cappell and Iacovone Jr. [12]	1999	0.486%	30	73.1	65% (129)	200	1% (2)	60% (119)	40% (79)	x	x	30% (59)
Al-Mallah et al. [13]	2007	0.722%	x	70.1	41% (9)	22	100% (22)	0	0	x	x	x
Lim et al. [14]	2013	x	30	65.0	49% (43)	87	0	79% (69)	21% (18)	x	x	x
Sayana et al. [15]	2014	x	x	74.0	54% (140)	258	7% (17)	76% (196)	17% (45)	x	x	8% (20)
Choudry et al. [16]	2009	x	x	x	x	9	100% (9)	0	0	x	x	x

*Total*		(274/56,674)	x	x	695	1178	235	705	238	102	60	183

*Average*		0.48%	27.1	71.3	59.0%	x	19.9%	59.8%	20.2%	32.9%	19.4%	18.2%

AMI; acute myocardial infarction (not specified); STEMI: ST elevation MI; NSTEMI: non-ST elevation MI; CHF: congestive heart failure.

^*∗*^Study divided based on ACS subtype.

^+^Incidence of endoscopy in all ACS patients.

“x”: not reported.

^1^All ACS.

^2^Unstable angina.

**Table 2:** Indications for endoscopy & type of intervention.

Indication for endoscopy								Intervention			
Author	Year	Hematemesis	Melena	BRBPR	GIB	Occult GIB	Other	Timing	Therapy	Sedation	Type
Cappell and Iacovone Jr. [3]	1996	0	0	0	0	0	100% (28)	22.3	100% (28)	x	PEG
Montalvo and Lee [4]	1996	0	0	0	88% (7)	0	12% (1)	9.6	x	x	EGD
Al-Ebrahim et al. [5]	2012	39% (18)	52% (24)	9% (4)	0	0	0	3	x	x	EGD
Nojkov and Cappell [6]	2010	0	0	0	0	0	100% (13)	6.9	100% (13)	100% (13)	ERCP
Nojkov and Cappell [6]	2010	0	0	0	0	0	100% (6)	7.5	83% (5)	100% (6)	ERCP
Mumtaz et al. [7]	2008	0	0	0	76% (65)	16% (14)	7% (6)	6	31% (26)	100% (85)	EGD
Spier et al. [8]	2007	0	0	0	65% (87)	13% (18)	22% (29)	7.5	19% (25)	100% (135)	Mult.
Lin et al. [9]	2006	33% (42)	43% (54)	6% (8)	0	10% (12)	8% (10)	7	17% (18)	x	EGD
Cappell [10, 11]	2004	0	5% (5)	35% (35)	0	47% (47)	13% (13)	15.5	15% (15)	86% (86)	CS
Cappell [10, 11]	2004	x	x	x	76% (59)	x	24% (19)	13	4% (3)	38% (30)	FS
Cappell and Iacovone Jr. [12]	1999	44% (88)	22% (43)	7% (13)	0	18% (36)	10% (20)	9.1	10% (19)	x	EGD
Al-Mallah et al. [13]	2007	0	0	0	100% (22)	0	0	3.4	x	x	EGD
Lim et al. [14]	2013	25% (22)	30% (26)	3% (3)	0	34% (30)	7% (6)	5.2	x	x	EGD
Sayana et al. [15]	2014	x	x	x	38% (98)	33% (86)	29% (74)	x	x	90% (232)	Mult.
Choudry et al. [16]	2009	0	0	0	89% (8)	0	11% (1)	12	11% (1)	x	EGD

*Total*		170	152	63	346	243	226	9.0	153	587	x

*Average*		14.2%	12.7%	5.3%	28.8%	20.3%	18.8%	x	20.2%	87.0%	x

BRBPR: bright red blood per rectum; GIB: gastrointestinal bleeding; ERCP: endoscopic retrograde cholangiopancreatography; CS: colonoscopy; FS: flexible sigmoidoscopy; EGD: esophagogastroduodenoscopy.

“x”: not reported; Mult.: several different endoscopic modalities.

**Table 3:** Endoscopic findings.

Endoscopic findings									
Author	Year	PUD	MW	Gastritis Esophagitis	Colitis	Varices	Malignancy	Other	Normal
Cappell and Iacovone Jr. [3]	1996	0	0	0	0	0	0	100% (28)	0
Montalvo and Lee [4]	1996	63% (5)	13% (1)	0	0	0	0	13% (1)	13% (1)
Al-Ebrahim et al. [5]	2012	78% (35)	0	4% (2)	0	14% (6)	2% (1)	2% (1)	0
Nojkov and Cappell [6]	2010	0	0	0	0	0	0	85% (11)	15% (2)
Nojkov and Cappell [6]	2010	0	0	0	0	0	0	83% (5)	17% (1)
Mumtaz et al. [7]	2008	42% (41)	0	28% (27)	0	20% (20)	0	0	10% (10)
Spier et al. [8]	2007	x	x	x	x	x	x	x	x
Lin et al. [9]	2006	30% (31)	7% (7)	25% (26)	0	1% (1)	3% (3)	15% (16)	20% (21)
Cappell [10, 11]	2004	0	0	0	20% (20)	0	8% (8)	18% (18)	54% (54)
Cappell [10, 11]	2004	0	0	0	21% (16)	0	4% (3)	13% (10)	63% (49)
Cappell and Iacovone Jr. [12]	1999	32% (64)	4% (7)	25% (49)	0	1% (4)	0	25% (49)	14% (27)
Al-Mallah et al. [13]	2007	x	x	x	x	x	x	x	x
Lim et al. [14]	2013	21% (18)	1% (1)	55% (48)	x	1% (1)	1% (1)	7% (6)	14% (12)
Sayana et al. [15]	2014	x	x	x	x	x	x	x	x
Choudry et al. [16]	2009	11% (1)	0	78% (7)	0	0	0	0	11% (1)

*Total*		195	16	159	36	32	16	145	178

*Average*		25.1%	2.1%	20.5%	4.6%	4.1%	2.1%	18.7%	22.9%

PUD: peptic ulcer disease; MW: Mallory-Weiss.

“x”: not reported.

**Table 4:** Outcomes of endoscopy & complication rates.

Outcomes						
Type	Total	Complication rate	Aborted	Surgery angiography^+^	Repeat	All-cause mortality^*∗*^
EGD	65	11.5%	1% (4)	2 % (14)	1% (4)	6% (33)
CS	9	9.0%	x	1% (1)	x	9% (9)
FS	2	2.5%	1% (1)	x	3% (2)	15% (12)
PEG	3	10.3%	0	x	3% (1)	0
ERCP	3	14.3%	0	0	10% (2)	5% (1)
ENDO	26	6.6%	1% (2)	0.3% (1)	0.3% (1)	0.3% (1)

*Total*	108	9.1% (108/1188)	2.0% (7/353)	3.6% (16/444)	2.2% (10/450)	8.1% (56/689)

ERCP: endoscopic retrograde cholangiopancreatography; CS: colonoscopy; FS: flexible sigmoidoscopy; EGD: esophagogastroduodenoscopy.

^*∗*^Deaths not attributable to endoscopy.

^+^Gastrointestinal surgery or angiography for further management following endoscopy.

“x”: not reported. ENDO: data from two publications: Spier et al. [8]and Sayana et al. [15], in which several endoscopic modalities were used.

**Table 5:** Complications by type.

Complications											
Type	Respiratory failure	Desaturation	Hypotension	ACS	Arrhythmia	Perforation	Death	Other	Not specified	Minor complication^*∗*^	Major complication^*∗*^
EGD	3% (2)	6% (4)	22% (14)	11% (7)	6% (4)	3% (2)	5% (3)	3% (2)	42% (27)	66% (43)	29% (19)
CS	0	0	78% (7)	0	11% (1)	0	11% (1)	0	0	89% (8)	11% (1)
FS	0	0	0	0	100% (2)	0	0	0	0	100% (2)	0
PEG	0	33% (1)	0	0	0	0	0	67% (2)	0	67% (2)	33% (1)
ERCP	0	0	67% (2)	0	0	0	0	33% (1)	0	0	100% (3)
ENDO	0	0	12% (3)	0	8% (2)	0	0	0	81% (21)	81% (21)	19% (5)

*Total* (*n* = 108)	2	5	26	7	9	2	4	5	48	76	29

*Percent*	1.9%	4.6%	24.1%	6.5%	8.3%	1.9%	3.7%	4.6%	44.4%	72.4%	27.6%

ERCP: endoscopic retrograde cholangiopancreatography; CS: colonoscopy; FS: flexible sigmoidoscopy; EGD: esophagogastroduodenoscopy; ENDO: data from two publications, Spier et al. 2007 [8] and Sayana et al. 2014 [15], in which several endoscopic modalities were employed.

^*∗*^Data on 105 events.

## Appendix

### Literature Search Adapted to Medline

1. Esophagoscopy/
2. Gastroscopy/
3. Colonoscopy/
4. Duodenoscopy/
5. ERCP/
6. Endoscopy, Digestive System/
7. sigmoidoscopy/
8. enteroscop$.tw.
9. EGD.ti,ab.
10. Endoscop$.ti,ab.
11. Gastroscop$.ti,ab.
12. Colonoscop$.ti,ab.
13. ERCP.ti,ab.
14. sigmoidoscop$.ti,ab.
15. Endoscopy, Gastrointestinal/
16. or/1–15
17. Myocardial Infarction/
18. Acute Coronary Syndrome/
19. angina/
20. Stemi/
21. nstemi/
22. (Myocardial adj Infarction).tw.
23. (Acute adj Coronary adj Syndrome).tw.
24. angina.ti,ab.
25. stemi.ti,ab.
26. nstemi.ti,ab.
27. or/17–26
28. 16 and 27
